# Brain-derived neurotrophic factor protects serotonergic neurons against 3,4-methylenedioxymethamphetamine (“Ecstasy”) induced cytoskeletal damage

**DOI:** 10.1007/s00702-022-02502-8

**Published:** 2022-04-14

**Authors:** F. Bavato, S. Stamatakos, C. M. Yde Ohki, E. Seifritz, P. Romualdi, E. Grünblatt, B. B. Quednow

**Affiliations:** 1 Department of Psychiatry, Psychotherapy and Psychosomatics, Psychiatric University Hospital Zurich, University of Zurich, Lenggstrasse 31, CH-8032 Zurich, Switzerland; 2 Department of Pharmacy and Biotechnology, University of Bologna, Bologna, Italy; 3 Department of Child and Adolescent Psychiatry and Psychotherapy, Psychiatric University Hospital Zurich, University of Zurich, Zurich, Switzerland; 4 Zurich Center for Integrative Human Physiology, University of Zurich, Zurich, Switzerland; 5 Neuroscience Center Zurich, University of Zurich and Swiss Federal Institute of Technology Zurich, Zurich, Switzerland

**Keywords:** Ecstasy, MDMA, Neurotoxicity, Neurofilament light chain, Brain-derived neurotrophic factor, Serotonergic neurons

## Abstract

3,4-Methylenedioxymethamphetamine (MDMA, “Ecstasy”) use has been linked to persistent alterations of the brain serotonergic (5-HT) system in animal and human studies, but the molecular underpinnings are still unclear. Cytoskeletal structures such as neurofilament light chain (NfL) are promising markers of drug-induced brain toxicity and may be involved in MDMA neurotoxicity. The brain-derived neurotrophic factor (BDNF) promotes the growth and sprouting of 5-HT neurons and its differential response to MDMA administration was suggested to mediate dose- and region-dependent 5-HT damage by MDMA. However, the role of BDNF pre-treatment in preventing MDMA neurotoxicity and the potential effects of MDMA on NfL are still elusive. Therefore, a differentiated 5-HT neuronal cell line obtained from rat raphe nucleus (RN46A) was treated in vitro with either MDMA, BDNF, MDMA + BDNF, or vehicle. Cell viability (measured by MTT) and intracellular NfL levels (immunocytochemistry assay) were reduced by MDMA, but partially rescued by BDNF co-treatment. Our findings confirmed that BDNF levels can influence MDMA-induced 5-HT damage, and support BDNF to be a crucial target for neuroprotective interventions of the 5-HT system. We also provide evidence on the sensitivity of NfL to MDMA neurotoxicity, with potential implications for *in-vivo* monitoring of drug-induced neurotoxicity.

**Supplementary Information:**

The online version contains supplementary material available at 10.1007/s00702-022-02502-8.

## Introduction

3,4-Methylenedioxymethamphetamine (MDMA, “Ecstasy”) is a synthetic ring-substituted amphetamine derivate, which holds psychoactive proprieties through direct and indirect serotonergic (5-HT) and noradrenergic mechanisms (Gudelsky and Yamamoto 2008; Hysek et al. 2011). Due to its entactogenic effects, including enhancement of emotional empathy and prosocial feelings, MDMA is widely used for recreational purposes, especially in the club scene (Hysek et al. 2014). While *in-vivo* evidence of MDMA neurotoxicity from human studies is still limited, MDMA administration was consistently shown to induce selective and long-term damage of 5-HT neuron in animal and *in-vitro* models (Green et al. 2003). In animal studies, MDMA-induced alterations were shown to be dose- and region-dependent, with higher susceptibility for hippocampus and midbrain regions (Martínez-Turrillas et al. 2006; Battaglia et al. 1991). Particularly, hippocampal and fronto-cortical changes have been suggested to underlie consistently shown memory impairments in chronic MDMA users (Gouzoulis-Mayfrank et al. 2003; Wunderli et al. 2017; Abad et al. 2016; Quednow et al. 2006). Environmental factors (e.g., social stress) have also been suggested to mediate the individual vulnerability to MDMA-induced cognitive impairment, but a clear understanding of this interaction is still lacking (García-Pardo et al. 2017).

The neurobiological underpinnings by which MDMA induces 5-HT damage are still unclear. It was postulated, that the massive and prolonged alterations of 5-HT signaling elicited by MDMA may impair the energetic cellular homeostasis and activate oxidative stress pathways in 5-HT neurons (Huether et al. 1997; Sprague et al. 1998; Shankaran et al. 2001; Yamamoto and Raudensky 2008). MDMA was also linked to altered expression of the brain-derived neurotrophic factor (BDNF), which serves as a neurotrophin by promoting the growth and sprouting of 5-HT neurons (Hemmerle et al. 2012). In particular, the differential response of BDNF expression to MDMA administration observed in distinct brain regions in rats was suggested to mediate region-dependent neurotoxicity (Martínez-Turrillas et al. 2006). Regional differences in BDNF response may also differently drive the long-term recovery of 5-HT axons after MDMA treatment (Ádori et al. 2010). Consequently, BDNF may be a potential molecular target for interventions directed to prevent or treat MDMA-induced brain toxicity. In this direction, BDNF administration with intracortical infusion was found to promote the regrowth of 5-HT axons in adult rats treated with a 5-HT-specific neurotoxin (p-chloroamphetamine) (Mamounas et al. 2000). On the other hand, reduced BDNF response, as observed under prolonged social stress exposure in different animal models, may increase the susceptibility to drug-induced cognitive impairments (García-Pardo et al. 2017; Miczek et al. 2011). However, the role of BDNF pre-treatment in preventing MDMA neurotoxicity was not investigated yet.

Persistent neural alterations after MDMA administration were not only shown at a synaptic level, but also involve axonal degeneration and impairment of cytoskeletal structures (Abad et al. 2016; García-Cabrerizo and García-Fuster 2015; Capela et al. 2009; Fischer et al. 1995; Petschner, et al. 2018; Ly et al. 2018). In particular, chronic exposure to MDMA was reported to decrease neurofilament proteins (NFs) in the hippocampus of adolescent and young adult rats (García-Cabrerizo and García-Fuster 2015). NFs are intermediate filaments exclusively expressed in neurons, which holds structural functions by maintaining radial growth of axons, but are also involved in neurotransmission and axonal transport (Yuan et al. 2017). A reduction of NFs in different brain areas was also observed after exposure to methamphetamine, cocaine, and morphine (Beitner-Johnson et al. 1992; Sanchez et al. 2003). Importantly, NFs are released in extracellular matrices during neuroaxonal damage and could now be detected in blood by new-generation immunoassay methods (Disanto et al. 2017). Consequently, NFs and particularly the neurofilament light chain (NfL) subunit recently emerged as promising *in-vivo* markers of active brain pathology in several neuropsychiatric disorders, including multiple sclerosis, neurodegenerative diseases, traumatic brain injuries, and depression (Barro et al. 2018; Khalil et al. 2018; Bavato et al. 2021). Our recent work also reported elevated NfL levels in subjects with ketamine dependence and in chronic cocaine users, confirming its sensitivity to drug-induced neurotoxicity (Liu, et al. 2021; Bavato, et al. 2022). Thus, NfL represents an ideal translational biomarker of drug-induced brain pathology, which could be applied in both preclinical and clinical settings.

In the current study, we investigated the effects of MDMA treatment on cell viability and NfL levels in a differentiated 5-HT neuronal cell line obtained from rat raphe nucleus (RN46A) and the potential protective role of BDNF treatment. RN46A line was selected as raphe nucleus in rat was shown to be sensitive to MDMA-induced toxicity and RN46A also express NfL (Mamounas et al. 2000; White et al. 1994). We hypothesized that MDMA administration will impair cell viability (measured using MTT assay) and reduce NfL levels (verified in immunocytochemistry assays) and that both effects will be at least partially prevented by BDNF treatment. Our findings may elucidate the interaction between BNDF and 5-HT neurotoxicity induced by MDMA and may provide evidence on sensitivity of NfL to MDMA administration.

## Methods

### Reagents

DMEM/F-12 (11,320,033, Gibco™), Fetal Bovine Serum (FBS) (16,000,036, Gibco™), poly-D-lysine (P0899, Sigma Aldrich), laminin (23,017,015, Gibco™), trypsin–EDTA 0.25% (25,200,056, Gibco™), neurobasal medium (21,103,049, Gibco™), B27 (17,504,044, Gibco™), PBS with calcium and magnesium (14,040,133, Gibco™), PBS, pH 7.4 w/o Calcium and magnesium (10,010,015, Gibco™), LookOut Mycoplasma PCR Detection kit (Thermofisher). D,l-MDMA.HCl (Lipomed) and BDNF (B3795, Thermofisher), Cell Proliferation Kit I (MTT) ( 11,465,007,001, Roche), MAP-2 (dilution 1:1000, 188 004 Synaptic Systems), anti-NfL antibody (2835, Cell signaling), DAPI (ab228549, abcam), Alexa Fluor® 647 AffiniPure Donkey Anti-guinea pig IgG 800X (dilution 1:1000, Jackson ImmunoResearch), goat anti-mouse IgG Alexa Fluor 555 (diluition 1:50, A-21422 TermoFisher), DAKO Fluorescence Mounting Medium (S3023, Agilent). For further information see Supplementary Material 1.

### Cell culture

Rat brain raphe nucleus RN46A cell line was obtained from the laboratory of Prof. Whittemore (Kentucky Spinal Cord Injury Research Center). The cells (passage 21–27) were maintained in culture in a 5% CO_2_ incubator at 33 °C in DMEM/F-12 (11,320,033, Gibco™) medium supplemented with 10% of FBS (16,000,036, Gibco™) in six-well cell culture plates (83.3920, Sarstedt). For new passages, at approximately 80% confluency, the cells were removed by adding Trypsin–EDTA (25,200,056, Gibco™). To induce differentiation, RN46A cells (10,000 cells/well) were seeded for 24 h with DMEM/F-12 in 96-well plate (Nunc 167,008, Thermofisher) coated with poly-D-lysine (P0899, Sigma Aldrich) and laminin (code 23,017,015, Gibco™). Then, the medium was changed with neurobasal medium (21,103,049 Gibco™) supplemented with 2% B27 (17,504,044, Gibco™) and after an adaptation period of 24 h at 33 °C, the temperature was shifted to 37 °C, to initiate the differentiation. All of the experiments were performed after 8 days of differentiation induction. The neurobasal medium was changed every 3 days. RN46A cells were negatively tested for mycoplasma contamination with the LookOut Mycoplasma PCR Detection kit (Thermofisher) (see Supplementary Material 2). The expression of 5-HT transporters in RN46A cells was not specifically tested in our study as it has been widely demonstrated in previous investigations (White et al. 1994; Fjorback et al. 2009; Rumajogee et al. 2006; Eaton et al. 1995).

### Cell viability assay (MTT)

BDNF (B3795, Thermofisher) and d,l-MDMA.HCl (Lipomed) were dissolved in sterile water to create a stock solution at a concentration of 2000 ng/mL and 20 mM, respectively. For each experiment, fresh stock solution was diluted in culture medium (supplemented by B27) to obtain the final concentration of MDMA (1.3 mM) and BDNF (100 ng/mL). The neurotoxicity and the 50% growth inhibitory concentration (IC_50_) of RN46A was determined after exposing RN46A differentiated cells to different concentrations of MDMA (0.25–0.5–1–1.5–2 mM) for 24 h and 48 h (see Supplementary Material 3). For the pre-treatment studies, the cells were pre-treated with 100 ng/mL BDNF 1 h prior to the MDMA treatment that was set to 1.3 mM (IC_50_ dose). For control experiments, sterile water (same volume as MDMA solution) was diluted in culture medium, to exclude buffer-associated effects. MTT assays were performed 24 h and 48 h after treatments following the Cell Proliferation Kit I (MTT) (11,465,007,001, Roche) manufacturer’s protocol. Briefly, after the treatments, the cells were incubated with the MTT solution (final concentration 0.5 mg/mL) at 37 °C for 4 h. Then, the solubilization buffer was added and the well plate was allowed to stay in the incubator overnight to ensure that the formazan crystals were dissolved. The optical density was measure at 570 nm with a reference wavelength of 670 nm, using the Mithras2 LB 943 Multimode Reader (Berthold Technologies). Cell viability was reported as a percentage of vehicle-treated controls. All experiments were performed at least in quadruplicates.

### Immunofluorescence analysis

For immunocytochemistry experiments, RN46A cells (10,000 cells/well) were seeded in 96-well plates (Nunc 96-well plates, 167,008, Thermofisher) coated with poly-d-lysine (0.05 mg/mL, Sigma Aldrich, P0899) and laminin (0.01 mg/mL, Gibco™, 23,017,015). After 8 days under differentiation conditions, the cells were pre-treated with 100 ng/mL BDNF or vehicle 1 h prior to 1.3 mM (IC_50_) of MDMA for 24 h and 48 h. Following the incubation times, the cells were washed with PBS and fixed with paraformaldehyde 4% for 20 min at room temperature (RT), washed three times with PBS and permeabilized with blocking buffer solution (0.1%of Triton X-100, 1% BSA in PBS). The cells were incubated with MAP-2 (dilution 1:1000), and NfL (dilution 1:50) overnight at 4 °C. After 3 × washing with PBS, cells were incubated with secondary Donkey anti-guinea pig IgG Alexa Fluor 647 800X (dilution 1:1000), Goat anti-mouse IgG Alexa Fluor 555 (dilution 1:50) and 4′,6-diamidino-2-phenylindole (DAPI, 1:100; Abcam) for 30 min at RT. Cells were washed 3 × with PBS and one drop per well of fluorescence mounting medium was added. Immunofluorescence was detected with the cellSens Dimension software (version 2.3) on an Olympus fluorescent microscope. Two independent experiments, performed in triplicate (five pictures per each well), were analyzed.

### Statistical analysis

All statistical analyses were performed using Prism version 7.0 (GraphPad, San Diego, CA, USA). Quantitative variables were tested for normal distribution using the Kolmogorov–Smirnov test. Group effects were analyzed by one-Way ANOVA followed by Tukey’s multiples comparison or Kruskal–Wallis followed by Dunn’s multiples comparison test. To normalize the data of treatment effects on RN46A 5-HT neurons, the mean value of the control condition from two independent experiments with similar OD values was used. Quantitative data were expressed as mean ± SD. Results with p < 0.05 were considered statistically significant.

## Results

### Effects of MDMA and BDNF on cell viability by MTT assay

**Fig. 1:**
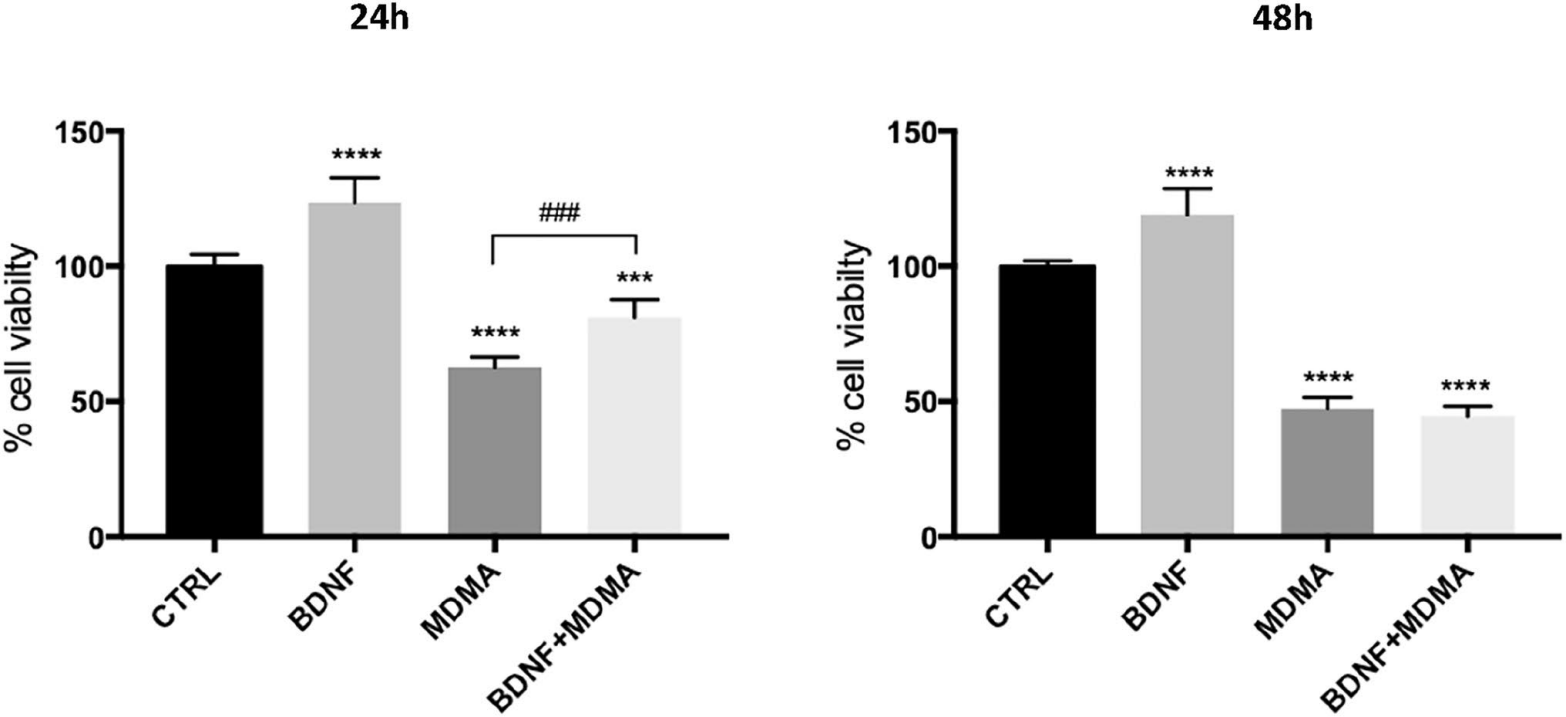
Effect of BDNF and MDMA treatment on RN46A serotonergic neurons viability. **A** Percentage of RN46A cell viability at 24 h. **B** Percentage of RN46A cell viability at 48 h. ANOVA followed by Tukey’s multiple comparison test: ***p < 0.001, ****p < 0.0001 compared to CTRL; ^###^*p* < 0.001, compared to MDMA. The values represent the mean ± SD of 3 independent experiments (*n* = 5/9)

Differentiation success was check under the microscope for morphological changes and using immunocytochemistry staining of MAP-2 marker, indicating mature neurons (see Supplementary Material 4). To determine the concentration of MDMA needed to cause 50% cell death/cell survival, differentiated RN46A cell line was exposed to different doses of MDMA for 24 h and 48 h. The IC_50_ was found to be 1.75 mM after 24 h and 1.15 mM after 48 h of the treatment (see Supplementary Material 4). To compare the effect on MDMA both at 24 h and 48 h, the concentration of MDMA was selected to be at 1.3 mM. The effect of BDNF on cell viability after MDMA was assessed using the MTT assay at 24 h and 48 h, utilizing 100 ng/ml of BDNF given 1 h prior 1.3 mM of MDMA. One-way ANOVA revealed significant differences between MDMA- and BDNF + MDMA-conditions compared to control at both 24 h (MDMA vs CTRL: F = 242.74, *p* < 0.001; BDNF + MDMA vs. CTRL: F = 32.47, *p* < 0.001) and 48 h (MDMA vs. CTRL: F = 824.94, *p* < 0.001; BDNF + MDMA vs. CTRL: F = 1071.25, *p* < 0.001), while a significant difference between BDNF + MDMA compared to MDMA-condition was only found at 24 h (24 h: F = 34.68, *p* < 0.001; 48 h: F = 2.20, *p* = 0.160) (Fig. 1). In particular, MDMA-induced reduction of cell viability was partially counteracted by concomitant BDNF administration at 24 h but not at 48 h.

### Effects of MDMA and BDNF on NfL by ICC analysis

**Fig. 2:**
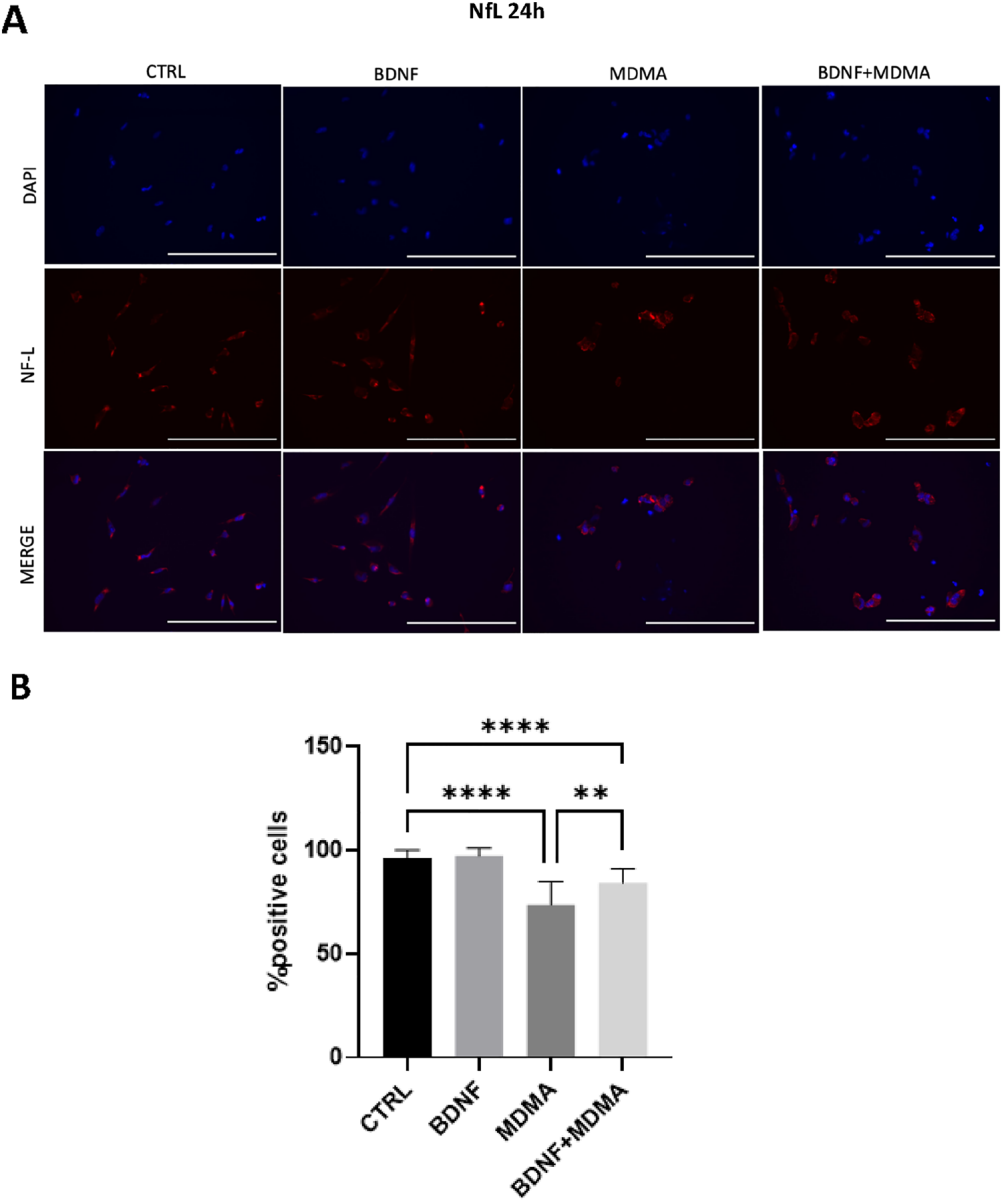
Alterations in NfL-positive 5-HT neurons after MDMA and protection via BDNF treatment at 24 h. **A** Exemplary immunofluorescence staining of NfL in RN46A differentiated cells at 24 h. NfL are stained red and cell nuclei are stained blue (DAPI). Scale bar: 200 μm. **B** The mean percentage of NfL-positive cells after 24 h treatment. The data represents mean ± SD (CTRL *n* = 27, BDNF *n* = 27, MDMA *n* = 17, BDNF + MDMA *n* = 15) and are analyzed by ANOVA followed by Tukey’s multiple comparison test. **p < 0.01, ****p < 0.0001

**Fig. 3:**
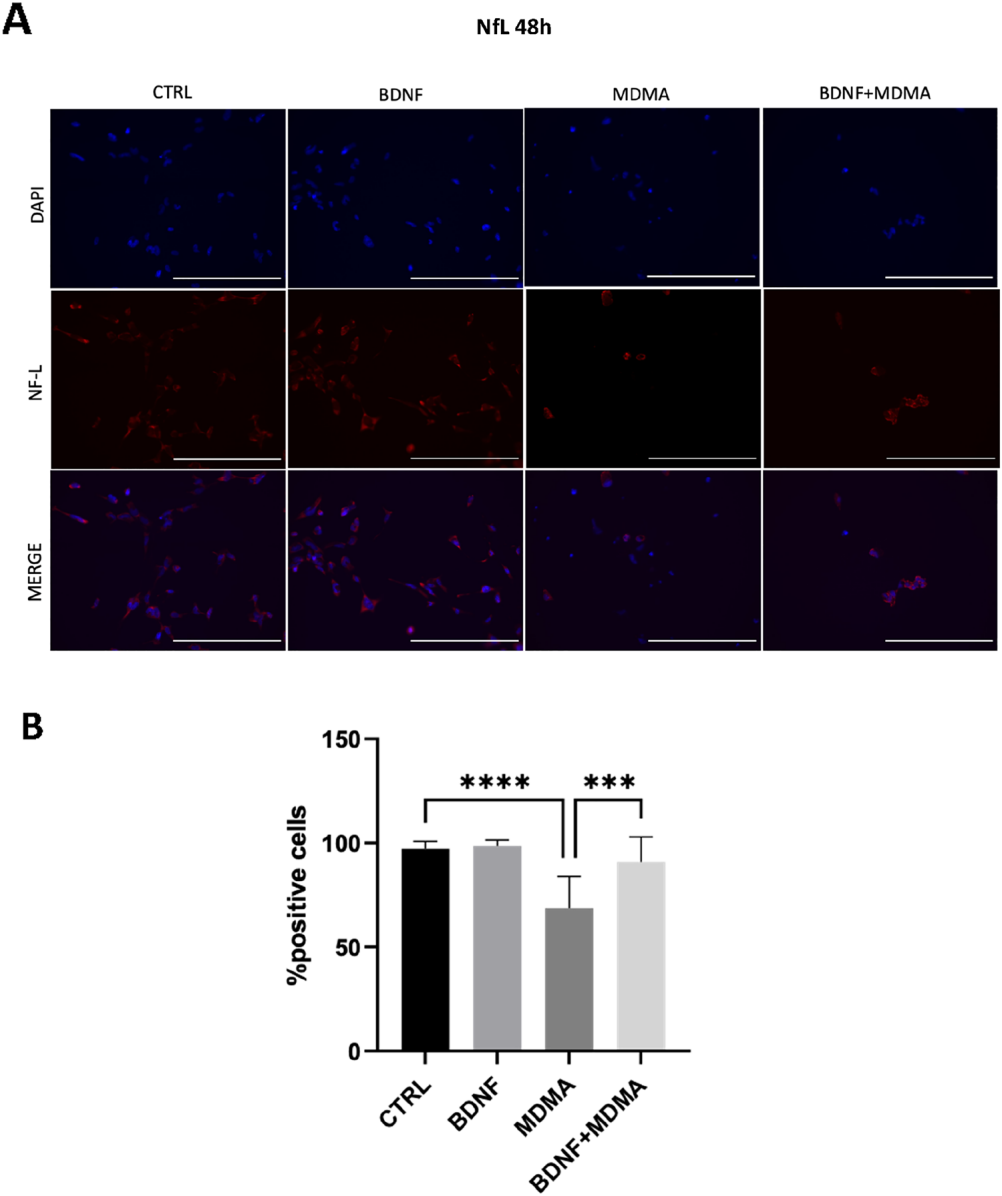
Alterations in NfL-positive 5-HT neurons after MDMA and protection via BDNF treatment at 48 h. **A** Exemplary immunofluorescence staining of NfL in RN46A differentiated cells at 48 h. NF-L are stained red and cell nuclei are stained blue (DAPI). Scale bar: 200 μm. **B** The mean percentage of NF-L-positive cells after 48 h treatment. The data represents mean ± SD (CTRL *n* = 29, BDNF *n* = 27, MDMA *n* = 16, BDNF + MDMA *n* = 25) and are analyzed by Kruskal–Wallis followed by Dunn’s test. ***p < 0.001, ****p < 0.0001

Two independent experiments, five pictures per well (3 wells per treatment), taken at 20 × magnification. The positive cells for NfL were counted manually and expressed in % relative to the total number of cells, assessed as DAPI positive cells. The immunocytochemistry assay revealed that the percentage of positive cell for NfL was significantly lower in MDMA and BDNF + MDMA-treated neurons compared to vehicle controls at 24 h (MDMA vs. CTRL: F = 92.84, *p *< 0.001; BDNF + MDMA vs. CTRL: F = 50.02, *p* < 0.001). Differently, the percentage of positive cell for NfL at 48 h was significantly lower in MDMA but not it BDNF + MDMA-treated neurons compared to vehicle controls (MDMA vs. CTRL: H(1) = 30.28, *p* = 0.001; BDNF + MDMA vs. CTRL: H(1) = 2.21, *p* = 0.137). Overall, the reducing effect of MDMA on NfL was counteracted by the pre-treatment with BDNF administration at both time-points (Figs. 2 and 3) (MDMA vs. BDNF + MDMA at 24 h: F = 9.95, p = 0.004; MDMA vs. BDNF + MDMA at 48 h: H(1) = 17.87, *p* < 0.001).

## Discussion

The main goals of the study were to clarify whether MDMA-induced toxicity on 5-HT neurons is rescued by BDNF treatment and to evaluate the sensitivity of NfL to MDMA-induced toxicity.

In the current investigation, a pre-treatment with BDNF partially prevented the decrease in cell viability of 5-HT neurons (RN46A) exposed to MDMA after 24 h. This finding is coherent with the literature on the protective functions of BDNF on 5-HT neurons (Homberg et al. 2014). Belonging to the neurotrophins family, BDNF supports the maintenance of monoaminergic and cholinergic neurons. A number of studies has demonstrated that reduced BDNF levels affect 5-HT signaling and the development of the serotonergic phenotype in neurons, but they also reported bidirectional interactions of 5-HT on BDNF levels (Homberg et al. 2014). In particular, the lack of 5-HT transporters in knockout rats, but also the low activity polymorphism of the 5-HT transporter in humans, were consistently linked to reduction of BDNF expression (Bhang et al. 2011; Molteni et al. 2010). It was also proposed that a negative spiral of low BDNF levels and impaired 5-HT signaling may lead to a reduction of brain resilience to environmental challenges, with crucial implications for neuropsychiatric disorders such as depression (Homberg et al. 2014). Similarly, different pattern of BDNF expression may also mediate individual vulnerability to MDMA-induced damage. Environmental stress (acute social defeat) has been shown to increase MDMA-induced cognitive impairment and depression-like behavior (García-Pardo et al. 2017). Alterations of BDNF in response to social stress have been also suggested to mediate individual vulnerability to other stimulant drugs (e.g., cocaine) (Miczek et al. 2011). BDNF may therefore represent a crucial pathway in determining individual differences in vulnerability for drug-induced neurotoxicity.

Not surprisingly, the strong modulation of 5-HT transmission by MDMA administration affects BNDF levels itself. Elevated BDNF levels in cortex but not in hippocampus were observed in rats treated with MDMA (Ádori et al. 2010). Moreover, high-affinity (i.e., Trk-B) and low-affinity (p75NTR) neurotrophin receptors were shown to be upregulated in the prefrontal cortex of rats treated with MDMA (Hemmerle et al. 2012; Wang et al. 2012). Considering these previous observations and our current findings, we suggest that BDNF elevation in specific brain regions can counteract MDMA-induced damage. On the contrary, the lack of a BNDF response in brain regions such as the hippocampus may explain their vulnerability to MDMA-induced toxicity. The absence of BDNF-related protective effects at 48 h may result from a saturation of this neuroprotective pathways over time. A prolonged exposition to MDMA could overwhelm the capacity of scavenging systems to protect the cellular homeostasis against oxidative stress reactions (Shankaran et al. 2001). In this direction, neural changes induced by MDMA were shown to be strongly variable with dosage regimen (Green et al. 2003).

The immunocytochemistry experiments confirmed a global decrease of NfL-positive neurons following MDMA treatment. Here, BDNF pre-treatment prevented NfL reduction at both 24 h and 48 h. This result suggest substantial neuroaxonal alterations after MDMA use and are in agreement with a previous study, in which NFs levels were decreased in rat hippocampus after chronic MDMA treatment (García-Cabrerizo and García-Fuster 2015). Other substances such as methamphetamine, morphine, and cocaine were also found to decrease NFs levels in different brain areas (Beitner-Johnson et al. 1992; Sanchez et al. 2003). The reduction of NfL induced by MDMA may also underlie the macroscopic alterations observed in MDMA-treated brain slices, which include marked reduction in 5-HT axonal density and damage of axon terminals (Capela et al. 2009). In knockout experiments, mice lacking NfL had severe inhibition of axon radial growth, nerve conduction and axonal regeneration, but also showed dysfunctions of synaptic transmission and altered hippocampus-dependent spatial memory (Yuan et al. 2017). Notably, impairment of hippocampus-dependent spatial learning was repeatedly reported in animals treated with MDMA and was linked to dysfunctional synaptic adaptations (Piper and Meyer 2004; Busceti et al. 2008). Memory dysfunctions were also the most consistent cognitive symptoms associated with MDMA use in humans (Gouzoulis-Mayfrank et al. 2003; Wunderli et al. 2017; Kalechstein et al. 2007). The protective effects of BDNF on NfL were even more consistent than in the cell viability experiments, and were still significant at 48 h. Thus, our findings support NfL to be a suitable translational marker to investigate MDMA-induced neuronal toxicity. Future investigations in animal models and in humans should now clarify, whatever the activation of neuroprotective pathways such as BDNF are sufficient to counteract MDMA-induced toxicity on NfL *in-vivo*.

Our investigation does not provide direct evidence on the underlying molecular pathways linking BDNF and the susceptibility to MDMA-induced neurotoxicity. Moreover, we only investigated a 5-HT cellular line from the raphe nucleus. The susceptibility of other neural populations to the interaction between MDMA/BDNF could strongly differ from our findings. However, considering the predominant involvement of 5-HT neurons in MDMA-induced neurotoxicity, we provided relevant evidence on its relationship to BDNF levels. Furthermore, we only focused on NfL levels and not on other axonal components that may also be affected by MDMA. Nonetheless, considering the increasing attention on NfL in neuropsychiatric research, this *in-vitro* model confirms its sensitivity to drug-induced neurotoxicity.

## Supplementary Information

Below is the link to the electronic supplementary material.

Supplementary file1 (DOCX 847 KB)

## Data Availability

The datasets generated during the current study are available from the corresponding author on reasonable request.
