# Supplementary material for: Brain-derived neurotrophic factor protects serotonergic neurons against 3,4-methylenedioxymethamphetamine (“Ecstasy”) induced cytoskeletal damage
Source: J Neural Transm (Vienna). 2022 Apr 14;129(5-6):703–11. doi: 10.1007/s00702-022-02502-8 (PMC9188522; doi:10.1007/s00702-022-02502-8)
Supplement: Supplementary file 1 — Supplementary file1 (DOCX 847 KB) [file 702_2022_2502_MOESM1_ESM.docx]

**Supplemental Material**

**Brain-derived neurotrophic factor protects serotonergic neurons against 3,4-methylenedioxymethamphetamine (“Ecstasy”) induced cytoskeletal damage**

*Bavato F., MD; ^1^* Stamatakos S., PhD; ^1,2^** , *Yde Ohki CM, MSc^3^,*  *Seifritz E., MD;^1^ Romualdi P., PhD; ^2^ Grünblatt E., PhD;^3,4,5^** Quednow B.B., PhD.^1,5^***

*^1^Department of Psychiatry, Psychotherapy, and Psychosomatics, Psychiatric Hospital, University of Zurich, Switzerland.*

*^2^Department of Pharmacy and Biotechnology, Alma Mater Studiorum University of Bologna, Italy.*

*^3^Department of Child and Adolescent Psychiatry and Psychotherapy, Psychiatric University Hospital Zurich, University of Zurich, Switzerland.*

*^4^Zurich Center for Integrative Human Physiology, University of Zurich, Switzerland.*

*^5^Neuroscience Center Zurich, University of Zurich and Swiss Federal Institute of Technology Zurich, Switzerland.*

***Supplementary Material 1: Reagents***

| **Reagent** | **Catalog number** | **Company** | **Final concentration**  **(if applicable)** | **Purpose** |
| --- | --- | --- | --- | --- |
| DMEM/F-12 medium | 11320033 | Gibco™ | - | Culture of RN46A cells before differentiation |
| Fetal Bovine Serum | 16000036 | Gibco™ | 10% in DMEM/F12 |  |
| Trypsin-EDTA 0.25% | 25200056 | Gibco™ | - |  |
| LookOut Mycoplasma PCR Detection kit | MP0035 | Sigma Aldrich | - | Verification of Mycoplasma-free RN46A culture |
| Neurobasal Medium | 21103049 | Gibco™ | - | Differentiation and culture of differentiated RN46A cells |
| B27 Supplement | 17504044 | Gibco™ | 2% in Neurobasal Medium |  |
| PBS with calcium and magnesium | 10010015 | Gibco™ | - | Coating for differentiated RN46A cells |
| Poly-D-Lysine | P0899 | Sigma Aldrich | 0.05 mg/mL |  |
| Mouse laminin | 23017015 | Gibco™ | 0.01 mg/mL |  |
| PBS, pH 7.4 without Calcium and magnesium | 10010015 | Gibco™ | - | General cell culture and immunocytochemistry |
| d,l-MDMA.HCl | MDM-94-HC | Lipomed | - | Treatment of differentiated RN46A cells |
| BDNF | B3795 | ThermoFisher | 100 ng/mL | Treatment of differentiated RN46A cells |
| Cell Proliferation Kit I (MTT) | 11465007001 | Roche | MTT final conc: 0.5mg/ml | Colorimetric assay for the quantification of cell viability |
| Anti-MAP-2 guinea pig antibody | 188 004 | Synaptic Systems | 1:1000 | Primary antibody for immunocytochemistry (Marker of mature neurons) |
| Anti-NfL mouse antibody | 2835 | Cell Signaling | 1:50 | Primary antibody for immunocytochemistry (Staining and quantification of NfL) |
| DAPI | ab228549 | Abcam | 1:100 | Immunocytochemistry (Nuclei staining) |
| Alexa Fluor® 647 AffiniPure Donkey Anti- guinea pig IgG 800X | 715-545-151 | Jackson ImmunoResearch | 1:1000 | Secondary antibody for immunocytochemistry |
| Alexa Fluor 555 goat anti-mouse IgG1 | A-21127 | ThermoFisher | 1:50 | Secondary antibody for immunocytochemistry |
| DAKO Fluorescence Mounting Medium | S3023 | Agilent | - | Enhancement of visualization of fluorescent samples after immunocytochemistry assays |

***Supplementary Material 2: Mycoplasma Contamination Assay***

LookOut® Mycoplasma PCR Detection kit was used to check a possible mycoplasma contamination in the cell line. The PCR products were run on a 1.2% agarose gel. Mycoplasma positive samples were expected to show bands in the range of 260 ± 8 bp and the positive control was expected to show a distinct band at 259 bp. A negative control showed a band at 481 bp, indicating a successful performance of the reaction, was present in every run. The absence of a band at 259 bp in the samples of supernatant of RN46A line confirmed the absence of mycoplasma (figure below).

**
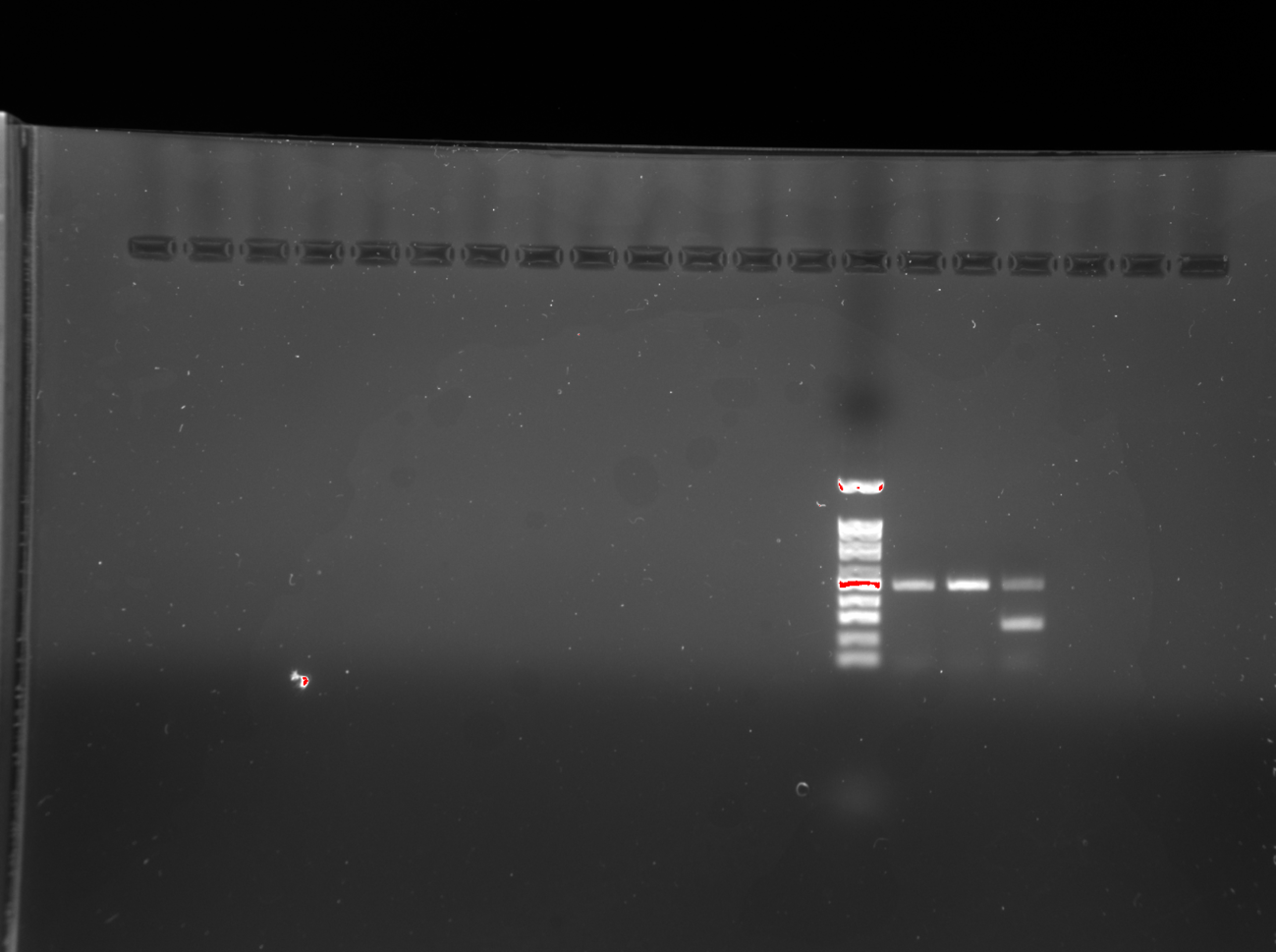
**

RN46A - +

100

500

1500

RN46A - +

***Supplementary Material 3: Cell viability curves***

To determine the concentration of MDMA needed to cause 50% cell death/cell survival, differentiated RN46A cell line was exposed to different doses of MDMA for 24h and 48h. Cell viability was determined using the MTT assay incubated for four hours. The IC_50,_ defined as the dose able to inhibit 50% of the cell growth, was found to be 1.75 mM after 24h and 1.15 mM after 48h. To compare the effect on MDMA both at 24h and 48h, the dose of MDMA used with 100 ng/ml BDNF pre-treatment was selected to be at 1.3 mM. Graphs representing % cell viability curves and IC_50_ values (concentration responsible for 50% cells growth inhibition) of RN46A differentiated cell line versus the concentration of MDMA are reported below. The values reported are the mean of two independent experiments (n=6 each experiment; Total n=12) ± SD.

***Supplementary Material 4: Differentiated RN46A cell line***

Exemplary immunofluorescence staining of differentiated RN46A cell line showing the expression of MAP-2 in green with cell nuclei marked in blue (DAPI). RN46A cells proliferating at 33 °C have a fibroblastic morphology, while at 37 °C, they cease dividing and take on a generally bipolar neuronal-like morphology. Scale bar: 200 μm.


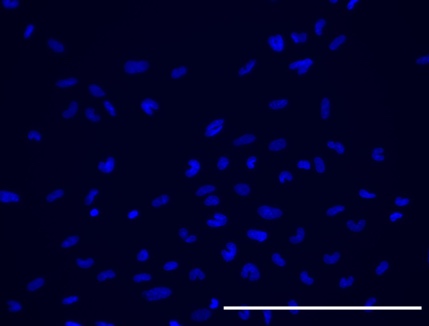

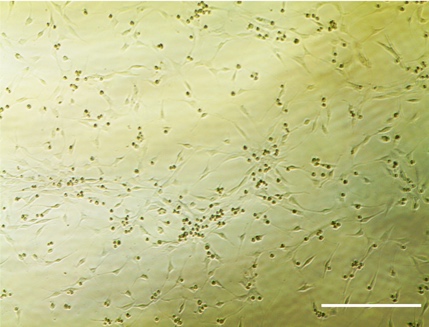


DAPI


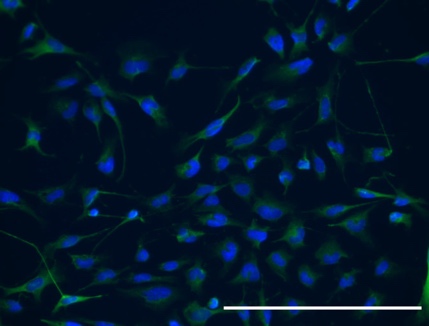

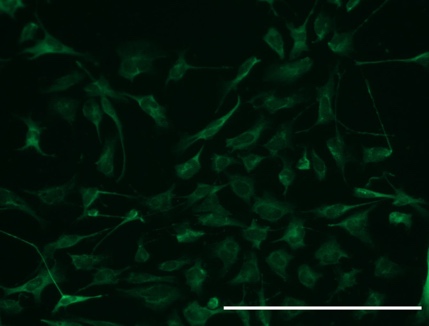


MERGE

MAP-2
